# Diseases of the Pancreas, and Their Surgical Treatment

**Published:** 1903-03

**Authors:** 


**Diseases of the Pancreas, and their Surgical Treatment.** By A. W. Mayo Robson, F.R.C.S., and B. G. A. Moynihan, M.S. (Lond.), F.R.C.S. Pp. 293. Philadelphia and London: W. B. Saunders & Company. 1902.

The surgery of the pancreas is one of the latest achievements of abdominal work, and the authors have taken a prominent part in bringing it to its present position. The book is, therefore, a timely record of what has been done in this direction, and though it may be needful to alter our present views on many points which require further elucidation, we are grateful for this volume which puts the known facts before us in a clear and masterly manner.

There have been many workers in this field, and the labours of Oser, Körte, and Opie have received recognition; but we failed to notice any reference to Riedel, who was among the first to point out the relationship between cholelithiasis and interstitial pancreatitis.

The greater part of the book is taken up by a description of the pathology, symptoms and treatment of the various forms of pancreatitis; and it is pleasant reading to find that there is hope even in such desperate cases as acute inflammation of the pancreas.

Pancreatic cysts are dealt with fully, taking up about one-fourth of the whole work; and the various interesting questions which these tumours afford are ably discussed.

Illustrative cases are given freely, and this is a part of the work which we think could be profitably curtailed in a future edition, as there is nothing gained by a repetition of cases almost similar in nature.

It does not appear to us desirable to place on the title page every small appointment held by the author of a book. In the present instance, we find a hospital mentioned which, according to the *Medical Directory*, contains only sixteen beds, and another hospital is given which we have failed to find mentioned at all in the *Directory* list of provincial hospitals.

To all who are interested in this branch of surgery we can strongly recommend this volume, which is likely to be the standard English text-book on the subject for some time to come.

